# Successful treatment of cytomegalovirus retinitis with oral/intravitreal antivirals in HIV-negative patients with lymphoma

**DOI:** 10.1038/s41433-022-02267-0

**Published:** 2022-10-03

**Authors:** Anastasia Tasiopoulou, Cristhian A. Urzua, Susan Lightman

**Affiliations:** 1 UCL/Institute of Ophthalmology, Moorfields Eye Hospital NHS Foundation Trust., London, UK; 2 Laboratory of Ocular and Systemic Autoimmune Diseases, Faculty of Medicine, University of Chile, Santiago, Chile; 3 Faculty of Medicine, Clínica Alemana Universidad del Desarrollo, Santiago, Chile

**Keywords:** Retinal diseases, Eye manifestations

## Abstract

**Objectives:**

To report patients with systemic lymphoma and cytomegalovirus (CMV) retinitis, treated with a combination of oral and intravitreal antiviral agents on an outpatient basis.

**Methods:**

Retrospective cases series. Information was gathered from the database of the Uveitis clinics at Moorfields Eye Hospital, United Kingdom from December 2014 to December 2018. The inclusion criteria comprised the diagnosis of systemic lymphoma, associated with a diagnosis of CMV retinitis. Exclusion criteria were alternative ocular diagnosis, human immunodeficiency virus (HIV), primary intraocular lymphoma, or other causes of immunosuppression.

**Results:**

All seven subjects had been under oncologist care for systemic lymphoma. CMV retinitis presented with a median of 61 months after the systemic lymphoma diagnosis. Five patients underwent a vitreous biopsy, and four of them returned PCR positive for CMV and the fifth patient had PCR positive in a blood sample. All patients were treated with oral Valganciclovir, with an induction dose of 900 mg every 12 h for up to 3 weeks until disease resolution and a maintenance dose thereafter. All but one received additional intravitreal Foscarnet injections, with a dose of 2.4 mg /0.1 ml.

**Conclusions:**

The management of patients with systemic lymphoma and CMV retinitis with oral and intravitreal antiviral agents, resulted in effective disease control.

## Introduction

Cytomegalovirus (CMV) represents a DNA herpesvirus which is highly prevalent worldwide [1, 2]. The global CMV seroprevalence is estimated to be 83% in the general population [3]. In immunocompromised patients, primary CMV infection can cause severe complications such as pyrexia, viremic-septicaemia and pneumonitis, with significant morbidity and mortality [4]. CMV retinitis is an uncommon condition which is classically presented in patients with human immunodeficiency virus (HIV), and it corresponds to an acquired immunodeficiency syndrome (AIDS) defining condition [5]. It is usually associated with CD 4 counts <50 cells/mm^3^ in patients with HIV [6, 7]. Nevertheless, sporadic cases of CMV retinitis in non-HIV subjects have been reported, particularly in patients with a known cause of immunodeficiency, such as immunosuppressive therapy, organ transplantation, post chemotherapy for cancer or inherited immunodeficiencies [8–10]. One possible mechanism that could be associated with the occurrence of CMV retinitis in non-HIV subjects is the exhaustion of T cells which are targeted by neoplasms and chemotherapies or radiotherapy [6, 7]. In addition, CMV retinitis has been reported in subjects with no evidence of underlying disorder [11]. These non-HIV cases are related to a myriad of causes of immunosuppression, and thus more heterogeneous clinical presentation and management have been described [8, 12, 13].

It is hypothesized that CMV virus spreads through the hematogenous route to the eye when the patient is immunosuppressed which disseminates to the retina resulting in retinitis. In the eye, the CMV primarily affects vascular endothelial cells and then the retinal pigment epithelium so that the virus is able to get access to the retinal tissue leading to retinal necrosis [1]. The standard treatment scheme consists of intravenous antiviral drugs, classically Ganciclovir, which requires hospitalization, with a scheme of induction and maintenance therapy, in order to achieve disease remission [4]. A body of evidence has been published regarding the role of intravitreal antiviral drugs, both as monotherapy (when systemic infection has been ruled out) and associated with systemic antiviral medication. In addition, Valganciclovir, an oral prodrug of Ganciclovir, has been shown as effective as Ganciclovir for CMV remission in HIV positive patients [14]. In the present study, we report a case series of non-HIV patients with a diagnosis of systemic lymphoma and CMV retinitis, treated with a combination of oral and intravitreal antiviral agents on an outpatient basis. These cases represent the largest case series of CMV retinitis in patients with systemic lymphoma.

## Methods

A retrospective case series of patients with the diagnosis of CMV retinitis associated with systemic lymphoma was conducted. Information was gathered from the database of the Uveitis clinics at Moorfields Eye Hospital (London, United Kingdom) from December 2014 to December 2018. The research protocol was approved by the Institutional Review Board and Ethic Committee of Moorfields Eye Hospital. The protocol fulfilled the tenants of the Declaration of Helsinki.

Only adults, with a minimum of follow-up of six months, were considered for this study. The inclusion criteria comprised the diagnosis of systemic lymphoma, associated with a diagnosis of CMV retinitis, according to the classification criteria published recently by the “Standardization of Uveitis Nomenclature (SUN) working group” [15]. Subjects were excluded if an alternative disease could explain the clinical manifestations diagnosed during the follow-up (e.g., toxoplasmosis, syphilis and acute retinal necrosis). Other exclusion criteria considered for the purpose of this study were: HIV, primary intraocular lymphoma (PIOL), or other causes of immunosuppression.

The following information was retrieved from the clinical records: age, gender, ethnicity, laterality, type of ocular manifestations, best corrected visual acuity (BCVA), laboratory investigations (including polymerase chain reaction (PCR) for CMV), ancillary testing (such as fundus photos, OCT, angiography), treatment type and duration, type of systemic lymphoma and its management, length of follow-up, clinical findings after antivirals initiation, and complications (such as cataract or glaucoma). Cataract was defined as any change in the normally clear media of the native lens that resulted in loss of vision. Lens changes reported as “trace” or “sclerosis” or with BCVA better than 20/40 were not included in the analysis [16, 17].

A positive response to treatment was defined as an improvement in the retinitis, vasculitis (clinically and on fundus fluorescein angiography—with resolution of the vascular leakage in involved areas of retina and no optic disc hyperfluorescence), and a two-step decrease in inflammation in anterior chamber and/or vitreous (although most of these CMV patients would not have had much anterior chamber cells or vitritis) or decreased level of inflammation to grade 0 in the anterior chamber and/or vitreous, as described by the SUN report published in 2005 [18].

BCVA cut-offs of 20/50 or less (low vision) and 20/200 or less (legal blindness) were used according to recommendations of SUN report [18]. In the descriptive statistics of the variables, we characterized the sample, calculating positional parameters and dispersion. Data were expressed as median (range), as sample size precludes the use of other dispersion and position measurements.

## Results

During the study period, seven adult patients (11 eyes) were diagnosed with lymphoma and CMV retinitis as defined above. Four out of them were female, with a median of age of 59 years and a range of 53–76 years. All subjects had been diagnosed with systemic lymphoma, and had been under oncologist care, with a median of time on cancer therapy of 9 months (range = 2–15 months). All patients were followed up for at least 6 months with a median of 11 months (range = 6–26 months).

At baseline ophthalmic evaluation, five of them were in remission, one patient was on chemotherapy, and one was waiting for bone marrow transplantation. CMV retinitis presented with a median of 61 months (range = 6–127 months) after the diagnosis of systemic lymphoma. Five patients underwent a vitreous biopsy, and four of them returned PCR positive for CMV and the fifth patient had PCR positive in a blood sample. Regarding the remaining 2 cases, 1 patient had bronchoscopy with their respiratory physician, which was positive for CMV and the other patient declined vitreous biopsy and the diagnosis of CMV retinitis was only clinical.

With regards to the investigations carried out for the differential diagnosis in these patients who did not have vitreous biopsy, they underwent widefield fundus photos (which showed whitish retinal infiltrates in the periphery with granular appearance), fundus fluorescein angiography (FFA) (which demonstrated hypofluorescence in the areas of retinal infiltrates and vascular leakage in the involved areas) and optical coherence tomography (OCT) in the areas of retinal infiltrates (which depicted full thickness retinal oedema). Furthermore, all seven patients underwent serologic antibodies for CMV. The CMV-IgM in serum was negative in 6 of our patients, whereas CMV-IgG was positive in all our patients. However, these titres have not been shown to be helpful in diagnosis  of CMV retinitis, as they reflect prior exposure [19].

All patients were treated with oral Valganciclovir, with an induction dose of 900 mg every 12 h for up to 3 weeks until disease resolution and a maintenance dose of 450 mg every 12 h thereafter. All but one received additional intravitreal Foscarnet injections, with a dose of 2.4 mg /0.1 ml intravitreally, as an induction therapy prior to oral treatment.

Five out of 6 patients, who received Foscarnet intravitreal injections, were given only one injection. Only one of these patients (Case 6) received more than one intravitreal injection and needed six injections for CMV disease control. This patient presented with left eye CMV retinitis and received the first 2 injections, as an induction therapy, 1 week apart and the rest 4 injections (1 week apart) 2 months later, due to recurrence of the retinitis. The decision to treat this female patient with an increased number of injections was based on the fact that her left eye (better seeing eye) BCVA at presentation was quite poor and the retinitis was fulminant threatening the optic nerve. Four patients (Case1, 2, 4 and 7) had bilateral CMV retinitis and were given one Foscarnet injection on both eyes.

Regarding the remaining patient who received only oral Valganciclovir and no intravitreal injections, the decision was based on the fact that the patient declined any further invasive diagnostic investigations and the diagnosis was only clinical.

Using this treatment approach, all patients had a good response to therapy as defined in methods. As shown in Table 1, ocular involvement was bilateral in four cases. Regarding inflammatory findings, three subjects presented with anterior chamber (AC) cells and vitreous involvement (either vitreous cells or haze on clinical examination), one patient only with AC cells and one only with vitreous haze. In addition, less common features were vasculitis (two subjects) and optic disc involvement (two subjects).

**Table 1:** Demographics and clinical features in seven patients with cytomegalovirus retinitis associated with systemic lymphoma.

Subjects	Type of lymphoma	Treatment for lymphoma	Laterality	Baseline BCVA	Last follow-up BCVA	Inflammatory findings at presentation	CMV retinitis type
Case 1^a^ (59 yo female)	T cell Lymphoma	Chemotherapy Interferon	BE	RE: 6/9 LE: CF	RE: 6/18 LE: 3/60	Vitreous haze/cells	Fulminant
Case 2^a^ (61 yo male)	Follicular Non-Hodgkin’s Lymphoma	Chemotherapy Rituximab	BE	RE: 6/6 LE: 6/24	RE: 6/6 LE: 6/6	Macular edema Optic nerve involvement Retinal vasculitis	Perivascular
Case 3 (64 yo male)	Non-Hodgkin’s Lymphoma	Bone marrow transplantation	LE	LE: 6/12	LE: 6/6	AC cells Vitreous haze/cells Optic nerve involvement	Granular
Case 4 (76 yo female)	Follicular Non-Hodgkin’s Lymphoma	Chemotherapy Radiotherapy Immunoglobulins	BE	RE: 6/6 LE: 6/9	RE: 6/6 LE: 6/9	AC cells Vitreous haze/cells	Granular
Case 5 (57 yo female)	T-cell Lymphoma	Chemotherapy Interferon	LE	LE: 6/18	LE: 6/18	AC cells	Granular
Case 6 (53 yo female)	Lymphoplasmacytic Lymphoma	Immunoglobulins	LE	LE: HM	LE: HM	AC cells Vitreous haze/cells Retinal vasculitis	Fulminant
Case 7 (52 yo male)	Diffuse large B-cell Lymphoma	Chemotherapy	BE	RE: 6/18 LE: 6/18	RE: 6/36 LE: 6/24	None	Granular

*BCVA* Best corrected visual acuity, *CMV* Cytomegalovirus, *yo* years old, *RE* right eye, *LE* left eye, *BE* both eyes, *AC* anterior chamber.

^a^Presented in the main text as illustrative cases.

Fulminant, granular and perivascular CMV retinitis were presented by two, four and one patients, respectively. Five out of eleven affected eyes presented with low vision at diagnosis, and two of them were severely visually impaired on presentation (BCVA: hand movement and count fingers, respectively). There was no significant change in visual acuities from baseline to last follow-up visit.

### Illustrative cases

#### Case 1

**Fig. 1:**
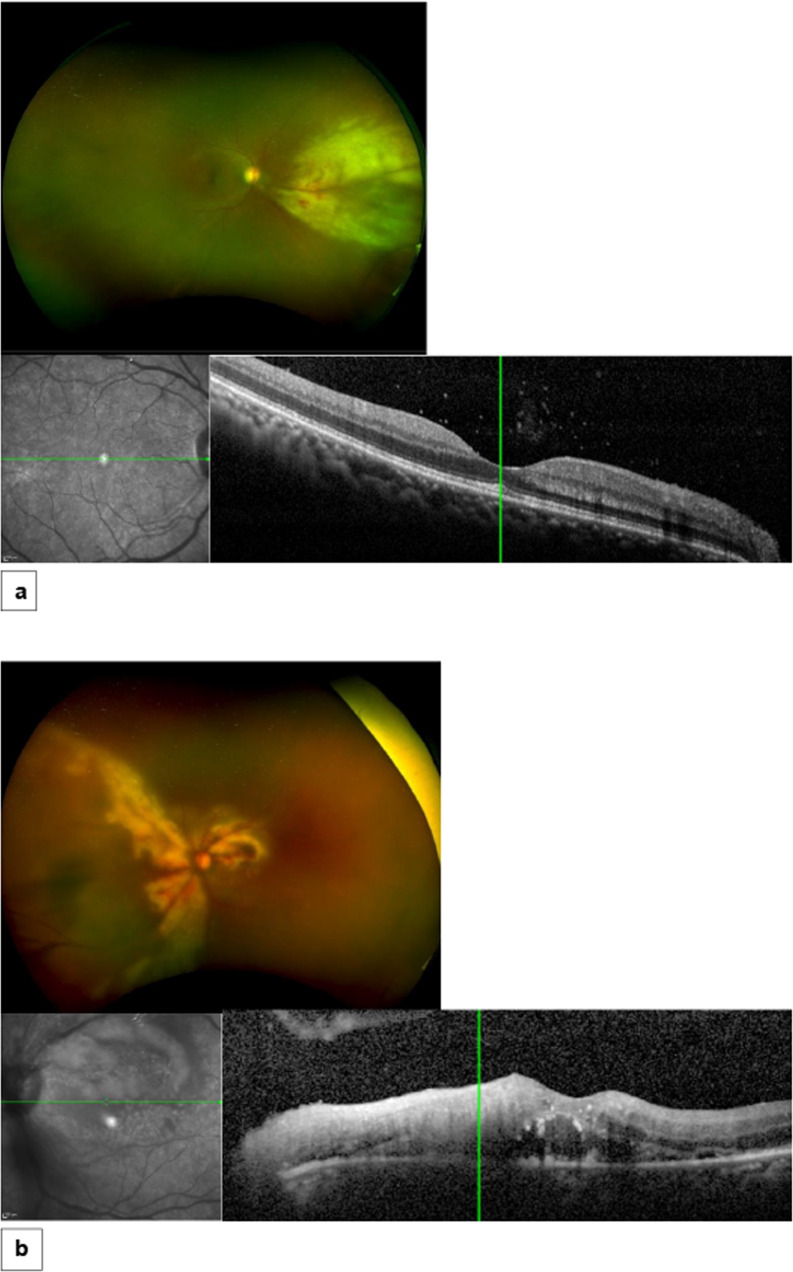
Illustrative case 1. Fundus photos, infrared images and optical coherence tomography (OCT) scans at presentation; **a** Right eye: Fundus photo shows confluent retinitis in the nasal retinal periphery, intraretinal haemorrhages, vascular sheathing and sclerotic arterioles. OCT shows vitreous cells, but normal inner and outer retina, **b** Left eye: Fundus photo demonstrates vitreous haze, retinal infiltrates and haemorrhages in the peripapillary area extending to the supratemporal, supranasal and infranasal quadrants. OCT depicts vitreous cells, full thickness retinal hyperreflectivity and deposits in the inner retina and subretinal area.

**Fig. 2:**
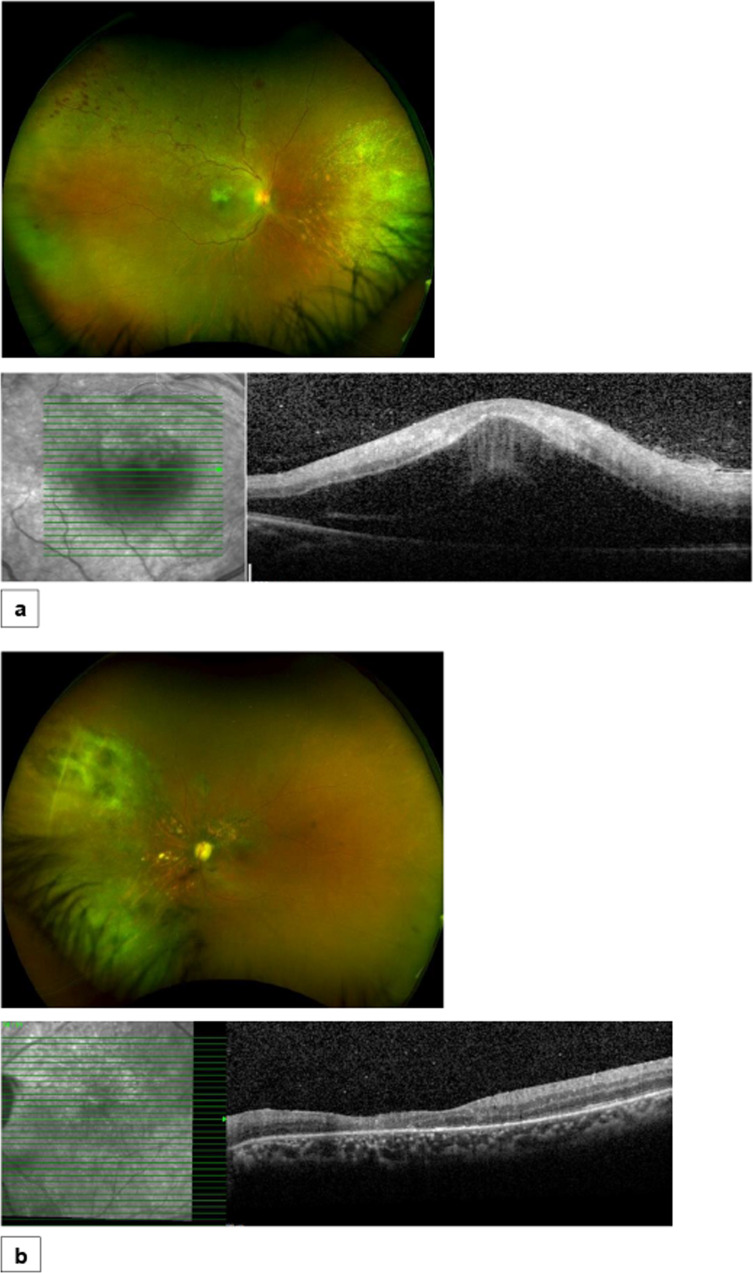
Illustrative case 1. Fundus photos, infrared images and OCT scans, 6 months after the initial presentation; **a** Right eye: Fundus photo shows resolved retinitis with sclerotic vessels in the nasal periphery and new intraretinal haemorrhages along the supratemporal arcade, consistent with new branch retinal vein occlusion (BRVO). OCT imaging shows significant cystoid macular oedema, secondary to BRVO, **b** Left eye: Fundus photo shows resolved retinitis with pigmented areas in the nasal periphery and atrophy peripapillary and along the supratemporal arcade and the posterior pole. OCT shows stable retinal atrophy in the posterior pole.

A 59-year-old female presented with a 5-day history of blurred vision in the left eye. She was diagnosed with adult T cell lymphoma of stage IVb secondary to HTLV (human T-lymphotropic virus type 1) infection, 6 months ago and was treated with chemotherapy and Interferon. At presentation, the lymphoma was on remission. On examination, her visual acuity, using the Snellen visual acuity chart, was 6/9 in her right eye and counting fingers (CF) at 10 cm in the left. The intraocular pressure was within normal limits and the anterior segment was unremarkable in both eyes. Fundus examination of the right eye detected an area of confluent retinitis in the nasal retinal periphery, about 2-disc diameters from the optic disc, with intraretinal haemorrhages, vascular sheathing and sclerotic arterioles. The fundus of the left eye had hazy media due to vitreous opacity and haemorrhagic retinal infiltrations in the peripapillary area extending to the supratemporal, supranasal and infranasal quadrants (Fig. 1). In view of the patient’s clinical presentation and medical history, the differential diagnoses were CMV retinitis, acute retinal necrosis and intraocular lymphomatous infiltration in both eyes. Diagnostic vitrectomy was performed in the left eye on the same day, which confirmed the presence of CMV DNA, no HSV or HZV DNA. CMV retinitis was clinically consistent with the type of fulminant - haemorrhagic necrosis. The patient was treated with an intravitreal injection of Foscarnet 2.4 mg/0.1 ml in both eyes, along with oral Valganciclovir 900 mg 12 hourly and Dexamethasone drops in both eyes. Valganciclovir in a dosage of 900 mg 12 hourly was administered for 3 weeks, followed by a maintenance dose of 900 mg daily for a period of 4 months. In liaison with the haematologist, intravenous Interferon was also administered. Over a 3-week period, there was gradual resolution of the retinitis in both eyes. However, in the left eye there was focal vitreous haemorrhage, but no retinal detachment in either eye. Over a period of 4 months, the CMV retinitis was in remission in both eyes and the topical and systemic treatment was discontinued. However, 6 months after the initial presentation, the patient was presented with an ischaemic branch retinal vein occlusion (BRVO) with secondary cystoid macular oedema in the right eye. FFA demonstrated an area of peripheral retinal nonperfusion in the supratemporal quadrant of the right eye and optic disc neovascularization (Fig. 2). Therefore, the patient was treated with sectoral retinal photocoagulation in the supratemporal quadrant of the right eye and a course of intravitreal anti-Vascular Endothelial Growth Factor (anti-VEGF) injections in the same eye. At her last follow-up, 1 year after the initial presentation, CMV retinitis was inactive in both eyes and the right eye BRVO was stable after the sectoral retinal photocoagulation and a course of 5 intravitreal anti-VEGF injections.

#### Case 2

**Fig. 3:**
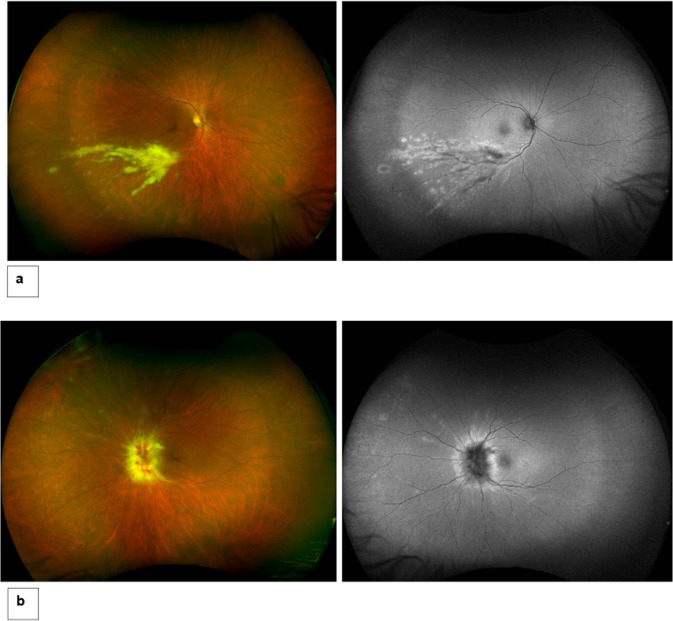
Illustrative case 2. Fundus photos and autofluorescent imaging (AF); **a** Right eye: Fundus image shows whitish retinal infiltrates along to the infratemporal retinal vein and artery, and their branches. In AF there are hyperautofluorescent lesions corresponding to the retinal infiltrates, **b** Left eye: Fundus photo demonstrates whitish infiltrate of the optic disc with haemorrhages and sheathing of the peripapillary retinal vessels with corresponding hyperautofluorescence in the AF.

A 61-year-old male presented with a 2-day history of blurred vision in the left eye. At presentation, his visual acuity was 6/6 in his right eye and 6/24 in the left, in the Snellen visual acuity chart. The intraocular pressure was within normal limits and there was no inflammation in the anterior chamber or the vitreous in either eye. Fundus examination of the right eye showed a localized area of whitish retinal infiltrates along to the infratemporal retinal vein and artery, and their branches. In the left eye there was whitish infiltrate of the optic disc with haemorrhages and sheathing of the peripapillary retinal vessels (Fig. 3). He was a known case of follicular non-Hodgkin’s lymphoma, which was diagnosed 6 years before presentation and managed with chemotherapy and Rituximab. Baseline blood tests showed chronic generalized hematologic problems, whereas other laboratory findings were normal, and an HIV test was negative. The patient’s clinical features were suggestive of CMV retinitis or lymphomatous infiltrates and left diagnostic vitrectomy performed on the same day. PCR analysis of vitreous fluid confirmed the presence of CMV DNA, no HSV or HZV DNA. Therefore, oral Valganciclovir 900 mg 12 hourly was administered for 3 weeks, followed by a maintenance dose of 900 mg daily for a period of 3 months. In addition, an intravitreal Foscarnet injection 2.4 mg/0.1 ml was administered in the left eye, due to optic disc involvement.

**Fig. 4:**
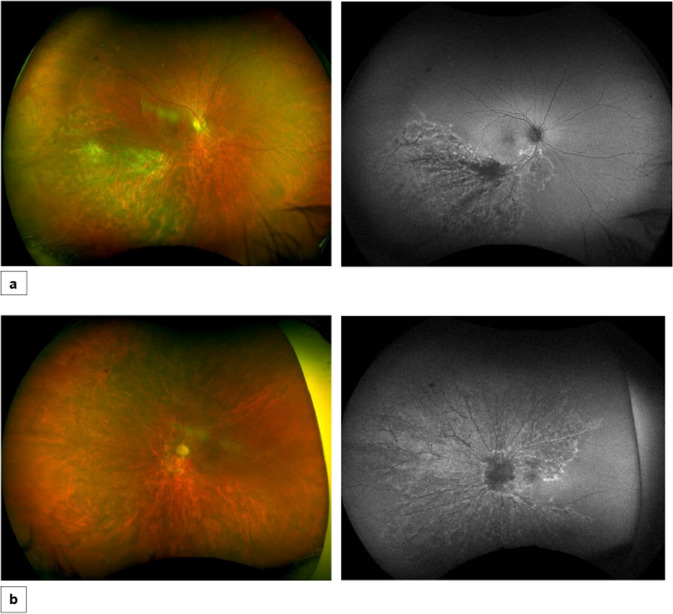
Illustrative case 2. Fundus photos and autofluorescence (AF) at last follow up; **a** Right eye: Fundus photo shows resolved retinitis with retinal atrophy along the infratemporal arcade depicted more clearly in AF, **b** Left eye: Fundus image depicts resolved retinitis with retinal atrophy more pronounced in AF.

The patient responded very well to treatment and 3 weeks later there was gradual resolution of the retinitis lesions in both eyes. At his last follow up, 26 months after the initial presentation, CMV retinitis was inactive bilaterally and the patient was discharged from the uveitis clinic (Fig. 4).

## Discussion

CMV retinitis represents a sight-threatening condition inserted in a clinical scenario characterized by the presence of an immunosuppressed state, secondary to HIV/AIDS or other conditions, such as immunomodulatory medications, cancer and diabetes, among others. In the present case series, CMV retinitis was presented in patients with systemic lymphoma. Special attention has to be taken into visual disturbances in lymphoma patients on treatment, since the eye has been described as a site for metastasis, and ocular complications—such as infections or paraneoplastic manifestations—could be presented in this subset of patients.

As described above, there are various presentations of CMV retinitis and the more prominent ones are the fulminant, the granular and the perivascular type. According to Ho et al., who studied retrospectively 35 eyes from 27 patients diagnosed with CMV retinitis with or without HIV infection, there was no significant difference in the prevalence of different types of retinitis between the HIV and the non-HIV patients. However, they concluded that the non-HIV patients presented more frequently with signs of intraocular inflammation such as vitritis, retinal arteritis, and vascular occlusions, compared to the HIV subjects [20]. Similar findings were described by Schneider et al. in 2013 and by Davis et al. in 2013, who described that CMV retinitis in non-HIV patients was associated with retinal arteriolar occlusions in elderly patients [12, 21]. These findings correspond to the ones from our case series, where 4 out of 7 patients (57%) had anterior segment inflammation at first presentation and the same percentage presented also with vitreous haze at the first visit (Table 1) and 2 out of 7 demonstrated retinal vasculitis either at first presentation or as secondary retinal vein occlusion. Thus, the diagnosis of infectious complications carries importance, since it determines the need of proper treatment in order to control the infection and thus to improve prognosis, which is better if early appropriate management is initiated [21–24]. In that sense, various antiviral treatment modalities have been used over the last decades. The most commonly used CMV retinitis treatment has been based on intravenous Ganciclovir in an inpatient setting. However, the disadvantages of the intravenous antiviral delivery include the necessity for hospitalization in a context of a prolonged treatment [13, 25]. Subsequently, a body of evidence has been shown the role of oral antivirals with or without adjunctive intravitreal therapy. Particularly, a randomized, controlled clinical trial published by Martin et al. in 2002, showed that the safety and efficacy of oral Valganciclovir is similar to the intravenous Ganciclovir when administered as induction therapy for CMV therapy in HIV patients [14, 26–28]. Valganciclovir is a prodrug of Ganciclovir and has been shown to have significantly higher oral absorption than Ganciclovir capsules. However, there are no publications for the use of oral Valganciclovir in the treatment of CMV retinitis in non-HIV patients. Interestingly, there are some case series published the previous years that showed that repetitive intravitreal Ganciclovir injections can be successful for the treatment of CMV retinitis in non-HIV patients [29, 30].

In the presented case series, an outpatient approach using oral plus intravitreal antivirals was applied in patients with a particular clinical context—systemic lymphoma—and all subjects presented an improvement in terms of inflammatory findings, which was set up as good response criteria. The rationale for the use of intravitreal Foscarnet was based on the fact that that in non-HIV patients, the intraocular inflammation is usually significant and more aggressive in contrast to HIV-related CMV retinitis, as mentioned above and in previous studies [20, 21]. Consequently, we decided to provide the patients with an induction therapy prior to the initiation of the effect of the oral treatment. The combination of oral and intravitreal treatment for CMV retinitis in non-HIV patients including the combination of intravitreal Foscarnet and oral Valganciclovir among others was also described by Iu et al., who reported the results of 20 eyes of 13 HIV negative patients (5 patients with lymphoma) with CMV retinitis treated effectively with oral and intravitreal therapy [31].

In this regard, in the analysis visual acuity was not considered as criteria for treatment response, since this important clinical variable could be particularly insensitive in patients with posterior uveitis in which chorioretinal lesions may cause structural irreversible damage [32–34]. Importantly, there is a need for PCR confirmation of the viral DNA, since retinitis lesions in this subset of patients may look different from that seen in HIV patients, due to the presence of more inflammatory findings, so can mimic alternatives diagnoses, in particular acute retinal necrosis. Therefore, if PCR results negative for CMV, an emphasis on ruling out other aetiologies should be the next step, with especial focus on syphilis, toxoplasmosis, and considering getting a vitreous sample for lymphoma.

It is important to mention that similarly to HIV patients with CMV retinitis, non-HIV patients with CMV retinitis occasionally may present Immune Recovery Uveitis (IRU). IRU is a non‐infectious uveitis related to immune recovery and defined as vitritis with significant floaters and a decline in vision with or without papillitis, macular changes (e.g. epiretinal membrane), proliferative vitreoretinopathy membranes and tractional retinal detachments. The treatment of IRU includes topical anti-inflammatory agents, periocular steroid injections, and oral anti-inflammatory agents. A dilemma can be encountered in the differential diagnosis between IRU and CMV retinitis because of the vitritis. This dilemma could be more difficult in cases of non-HIV patients compared to HIV positive subjects, since vitritis could be more prominent in the former ones. However, the presence of active retinitis in all our cases helped in distinguishing from an IRU even in the presence of vitritis.

Finally, discontinuation of the antiviral therapy deserves especial mention. In that sense, in HIV-positive patients with CMV retinitis, current guidelines include a sustained rise in CD4 + cells >100 cells/μL (3 to 6 months) plus no evidence of activity in ocular lesions [34, 35]. Also, several studies such as the ones of Jabs et al. in 2005 and Freeman et al. in 1996, suggested that CMV viral load in blood could be used as a screening tool to monitor HIV patients for reactivation and response to systemic treatment [36, 37]. Similarly, for non-HIV patients, Iu et al. reported that the systemic treatment was discontinued based on the CMV viral load in the blood and the presence of toxicity [31].

In our patients, the decision for discontinuation of antiviral therapy was based on the ocular disease inactivity and the resolution of the related CMV viraemia. In contrast to HIV-related CMV retinitis, which is strongly associated with the level of CD 4 count, in non-HIV patients with CMV retinitis, CD4 count is an unreliable prognostic factor. Based on this consensus, our proposal is to maintain therapy until no evidence of activity on clinical examination and ancillary testing is found, and a stable predisposing disease (i.e., systemic lymphoma) for 3 to 6 months is confirmed by the treating oncology team. In addition, in view of the high rate of recurrences in these patients after discontinuation of the treatment (33,3% of 20 eyes of HIV negative patients with CMV retinitis recurred in a mean interval of 6.4 weeks after cessation of treatment), there is need for regular retinal assessment of these patients [31].

To our knowledge, this report included the largest case series on CMV retinitis in patients with systemic lymphoma published at date. Importantly, an outpatient approach was presented, including a management with oral antivirals and intravitreal injections, which resulted in effective disease control. In that sense, no patient needs a switch to classical treatment with intravenous antivirals due to neither therapy failure nor adverse events.

## Summary

### What was known before

- Cytomegalovirus (CMV)-retinitis may be a complication in HIV-negative patients with lymphoma.

### What this study adds

- Oral/intravitreal antivirals presents as a successful treatment option of CMV retinitis in lymphoma patients

## Data Availability

The datasets generated during and/or analysed during the current study are available from the corresponding author on reasonable request.
